# Early Prediction of Breast Cancer Recurrence for Patients Treated with Neoadjuvant Chemotherapy: A Transfer Learning Approach on DCE-MRIs

**DOI:** 10.3390/cancers13102298

**Published:** 2021-05-11

**Authors:** Maria Colomba Comes, Daniele La Forgia, Vittorio Didonna, Annarita Fanizzi, Francesco Giotta, Agnese Latorre, Eugenio Martinelli, Arianna Mencattini, Angelo Virgilio Paradiso, Pasquale Tamborra, Antonella Terenzio, Alfredo Zito, Vito Lorusso, Raffaella Massafra

**Affiliations:** 1 Struttura Semplice Dipartimentale di Fisica Sanitaria, I.R.C.C.S. Istituto Tumori “Giovanni Paolo II”, Viale Orazio Flacco 65, 70124 Bari, Italy; mariac.comes@libero.it (M.C.C.); v.didonna@oncologico.bari.it (V.D.); p.tamborra@oncologico.bari.it (P.T.); r.massafra@oncologico.bari.it (R.M.); 2 Struttura Semplice Dipartimentale di Radiologia Senologica, I.R.C.C.S. Istituto Tumori “Giovanni Paolo II”, Viale Orazio Flacco 65, 70124 Bari, Italy; d.laforgia@oncologico.bari.it; 3 Unità Operativa Complessa di Oncologia Medica, I.R.C.C.S. Istituto Tumori “Giovanni Paolo II”, Viale Orazio Flacco 65, 70124 Bari, Italy; f.giotta@oncologico.bari.it (F.G.); a.latorre@oncologico.bari.it (A.L.); vitolorusso@me.com (V.L.); 4 Interdisciplinary Center for Advanced Studies on Lab-on-Chip and Organ-on-Chip Applications (ICLOC), University of Rome Tor Vergata, 00133 Rome, Italy; martinelli@ing.uniroma2.it (E.M.); mencattini@ing.uniroma2.it (A.M.); 5 Dipartimento di Ingegneria Elettronica, Università di Roma Tor Vergata, Via del Politecnico 1, 00133 Roma, Italy; 6 Oncologia Medica Sperimentale, I.R.C.C.S. Istituto Tumori “Giovanni Paolo II”, Viale Orazio Flacco 65, 70124 Bari, Italy; a.paradiso@oncologico.bari.it; 7 Unità di Oncologia Medica, Università Campus Bio-Medico, 00128 Roma, Italy; a.terenzio@unicampus.it; 8 Unità Operativa Complessa di Anatomia Patologica, I.R.C.C.S. Istituto Tumori “Giovanni Paolo II”, Viale Orazio Flacco 65, 70124 Bari, Italy; a.zito@oncologico.bari.it

**Keywords:** DCE-MRI, neoadjuvant chemotherapy, breast cancer recurrence, Support Vector Machine, convolutional neural networks, transfer learning

## Abstract

**Simple Summary:**

An early prediction of Breast Cancer Recurrence (BCR) for patients undergoing neoadjuvant chemotherapy (NACT) could better guide clinicians in the identification of the most suitable combination treatments for individual patient scenarios. We proposed a transfer learning approach to give an early prediction of three-year BCR for patients undergoing NACT, using DCE-MRI exams from I-SPY1 TRIAL and BREAST-MRI-NACT-Pilot public databases. Because no technical expertise is required in the extraction of meaningful features from images, the predictive model qualifies as a user-friendly tool for any medical expert in support of therapeutic choices. Only pre-treatment and early-treatment MRI examinations were analyzed to allow for potential therapy changes at a very early stage of treatment. We tested the strength of the model on an independent test. The best predictive performances (accuracy of 85.2%, sensitivity of 84.6%, and AUC of 0.83) were achieved by combining the extracted features with some clinical factors: age, ER, PgR, HER2+.

**Abstract:**

Cancer treatment planning benefits from an accurate early prediction of the treatment efficacy. The goal of this study is to give an early prediction of three-year Breast Cancer Recurrence (BCR) for patients who underwent neoadjuvant chemotherapy. We addressed the task from a new perspective based on transfer learning applied to pre-treatment and early-treatment DCE-MRI scans. Firstly, low-level features were automatically extracted from MR images using a pre-trained Convolutional Neural Network (CNN) architecture without human intervention. Subsequently, the prediction model was built with an optimal subset of CNN features and evaluated on two sets of patients from I-SPY1 TRIAL and BREAST-MRI-NACT-Pilot public databases: a fine-tuning dataset (70 not recurrent and 26 recurrent cases), which was primarily used to find the optimal subset of CNN features, and an independent test (45 not recurrent and 17 recurrent cases), whose patients had not been involved in the feature selection process. The best results were achieved when the optimal CNN features were augmented by four clinical variables (age, ER, PgR, HER2+), reaching an accuracy of 91.7% and 85.2%, a sensitivity of 80.8% and 84.6%, a specificity of 95.7% and 85.4%, and an AUC value of 0.93 and 0.83 on the fine-tuning dataset and the independent test, respectively. Finally, the CNN features extracted from pre-treatment and early-treatment exams were revealed to be strong predictors of BCR.

## 1. Introduction

Neoadjuvant chemotherapy (NACT) is being increasingly used as the frontline therapy for the management of high-risk, locally advanced or unresectable breast cancers, improving the eligibility for breast conserving surgery to be performed by shrinking larger tumors [1,2,3]. The care of Breast Cancer (BC) patients has changed considerably over the last few decades, integrating NACT into the multidisciplinary management of many neoplasms. The main objective of NACT has been for a long time to obtain the downstaging of the neoplasm, increasing the number of women eligible for breast conservation surgery. A positive prognostic impact and a favorable response to NACT have been shown for some tumors, including those in non-advanced stages, such as triple negative and HER2+ breast cancers. However, the type of cancer staging at diagnosis and the pathological response obtained through NACT greatly influence the patient’s prognosis and the possibility of disease relapse [4]. One key benefit of NACT consists of the observation of how the tumor changes or evolves during treatment. Some research studies have underlined that the treatment efficacy can already be evaluated through the exams acquired after the early stages of the NACT, or even only after its first cycle [5,6,7,8,9]. In particular, the inclusion of dynamic contrast-enhanced magnetic resonance imaging (DCE-MRI) in the NACT protocol yields the possibility to identify finer tumor properties that, beyond structural changes in tumor size and shape, could serve as potential earlier indicators of patient Recurrence-Free Survival (RFS) [10,11]. The term RFS conventionally indicates the period of time after the beginning of treatment for a cancer that the patient survives without any signs or symptoms of that cancer. In clinical experimentations, RFS can be measured to evaluate the efficacy of a new treatment [12,13]. In this paper, we focused on Breast Cancer Recurrence (BCR), i.e., the recurrence event identified as local or distant progression or death within a defined interval of time [13]. An accurate prediction of RFS or BCR at early stages of the NACT regimen could be useful to improve clinical choices, especially for patients subject to high risk of recurrence. Such patients could be prioritized for active surveillance or more aggressive therapeutic options, thus reducing the risks of ineffective or unnecessary treatment. For example, potential therapy changes at a very early stage of treatment, such as dose-dense neoadjuvant chemotherapy [14], or a combination of chemotherapy and other drugs, such as trastuzumab [15], can be taken into account. Otherwise, a reduced pre-operative waiting time can be considered.

In this context, the need for models based on image processing for early recurrence prediction is urgent. Towards this end, despite much progress being made, many approaches developed to date still suffer from important limitations. Radiomic analysis using regression models with handcrafted features represents the most common choice in RFS prediction [16,17,18]. Such types of models often make use of clinical measurements as well as radiomic features designed by data scientists [17,18,19]. Only a few classification approaches, always based on handcrafted radiomic features, have been freshly developed to predict the probability of BCR [11]. Despite the encouraging results achieved, handcrafted radiomic features consist of a limited set of pre-defined features based on human knowledge and are also affected by human bias.

In this study, we evaluated the crucial role of MR imaging in the early prediction of BCR in patients undergoing NACT under a novel perspective based on deep learning [20] and more specifically on transfer learning [21].

Deep learning is a quite recent machine learning framework based on the exploitation of artificial neural networks composed by interleaved layers to solve challenging imaging problems. The main power of deep learning consists in the possibility to extract a huge number of unperceivable-to-human features from raw images. Features of different level of abstraction can be extracted in a completely unsupervised manner: low-level features, e.g., edge and dots, and high-level features, e.g., shapes and objects. In particular, pre-trained and customized Convolutional Neural Networks (CNNs) have been shown to be of great promise. Basically, pre-trained CNNs are networks able to acquire knowledge from a huge number of natural (non-medical) images and transfer it onto commonly used images belonging to multiple domains of interest, including MRI data analysis. When such types of networks are applied to the task under study for feature extraction only, without classification, they do not require any training phase. This technique is also referred to as transfer learning. These networks are then combined with a standard machine learning classifier. Customized CNNs, however, are networks built to address a specific task. Because they are used to feature extraction and classification, a training phase, which can be computationally expensive, is required. The training and test images belong to the same application field. Pre-trained or customized CNNs have been successfully tested on areas of pathology and radiology, including breast mass detection [22] or classification [23]. Their usage has also been extended to predict recurrence of prostate cancer [24] or giant cell bone tumors [25]. Within the NACT framework on BC patients, CNN-based models have been proposed to predict the final response to therapy [26,27]. Despite these successes, the capability of CNNs to predict BCR for patients undergoing NACT has not yet been explored. To the best of our knowledge, this work represents the first attempt that utilizes CNN-extracted features and, in particular, transfer learning for an early prediction of recurrence for BC patients undergoing NACT.

In more detail, we proposed a hybrid machine learning-deep learning approach to predict the three-year BCR for patients from I-SPY1 TRIAL and BREAST-MRI-NACT-Pilot public databases. Through a pre-trained CNN, low-level features were extracted from the first post-contrast MRI examinations acquired prior to NACT and after its first cycle (pre-treatment and early-treatment exams, respectively). The huge number of extracted features was reduced by two combined feature selection methods. A set of the most stable features was identified and then used to build a standard machine learning classifier to discern patients that showed recurrence or not. The investigation of feature stability in combination with transfer learning can be recognized as a novel aspect of the work. The strength of the method was tested on an independent set of patients not involved in the feature selection process.

To better explain the main contribution of this work within the medical community, it is worthwhile to underline that breast RFS have been demonstrated to be modestly but significantly associated to pathological complete response (pCR) to neoadjuvant chemotherapy [16,28,29], where, with the term pCR, we mean the absence of residual invasive disease or lymph nodes at the end of the entire course of the therapy. Some research studies have highlighted how the overall effect of the treatment (in terms of pCR prediction) can already be accurately estimated after the first cycle of NACT [5,6,7,8,9]. Under such consideration, analyzing pre-treatment and early-treatment MRI exams allows us to combine tumor characteristics at the initial time of diagnosis and thus independently of any therapy with those after the first cycle of NACT, which can arguably give us an indication of the effectiveness of therapy.

The purpose of the work is to supply a completely automatized support tool that could aid medical figures to evaluate “early on” the efficacy of NACT before the end of the therapy itself, in order to better guide their therapeutic choices in accordance with the estimated probability of recurrence.

As a result of our investigation, the unperceivable-to-human features that were automatically measured by MRIs were revealed to be strong early predictors of BCR. Encouraging results have been obtained from the analysis of pre-treatment exams alone. Merging information extracted from pre-treatment and early-treatment MRI scans benefited predictive performances. Finally, a further improvement has been gained after augmenting the model with clinical variables such as age, estrogen receptor (ER), progesterone receptor (PgR), and human epidermal growth factor receptor 2 positive (HER2+).

Among clinical variables, we did not consider pCR because it is commonly evaluated at the end of the chemotherapy and after the surgery. The inclusion of pCR would have meant that the therapy had been completed. Instead, the proposed predictive tool wants to potentially enable changes to therapy at a very early stage of treatment and thus before surgery [10]. The usability of the software is addressed to a great number of medical figures given that a recommendation for neoadjuvant therapy ideally involves a multidisciplinary team, including experts in medical oncology, surgery, histopathology, radiology, and radiation oncology.

## 2. Materials and Methods

### 2.1. Data Selection and Description

**Figure 1:**
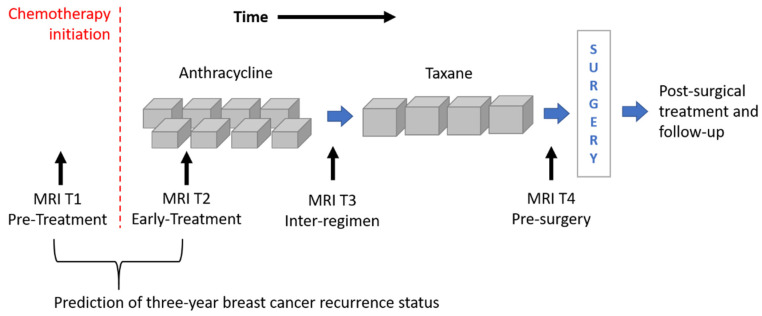
Outline of the MRI exams acquired for patients of the I-SPY1 TRIAL dataset undergoing neoadjuvant chemotherapy. MRIs related to timepoints T1 and T2 were analyzed for the prediction of three-year breast cancer recurrence.

The image dataset consisted of a set of DCE-MRIs from two public databases: the multi-site Investigation of Serial Studies to Predict Your Therapeutic Response with Imaging and molecular Analysis (I-SPY1 TRIAL) [1,16,30] dataset and the BREAST-MRI-NACT-Pilot dataset [31,32]. Both the databases are available on The Cancer Imaging Archive [33]. Currently, the I-SPY1 TRIAL dataset contains cases of 230 women with breast tumors of at least 3 cm in size who received NACT with an anthracycline-cyclophosphamide (AC) regimen alone or followed by a taxane. MRI examinations were performed using 1.5 T field-strength MR imaging systems [1,31] and consisted of a single pre-contrast image and another two images acquired approximately 2 ½ minutes and 7 ½ minutes post-contrast injection, respectively. Each patient counted four MRI scans at most (Figure 1): at around four weeks before the start of the treatment (MRI T1); at early treatment, i.e., at least two weeks after the first AC cycle and before the second AC cycle (MRI T2); at the end of the AC cycle and the start of taxane treatment if taxane was administered (MRI T3); and at the end of the entire chemotherapy cycle and prior to surgery (MRI T4).

The BREAST-MRI-NACT-Pilot dataset instead contains DCE-MRI exams of 64 patients undergoing NACT with stage II or III locally advanced breast cancer. The NACT regimen and the image acquisition respond to the same criteria of the I-SPY1-TRIAL database.

Two selection criteria were combined to identify a subset of patients from both datasets. First of all, we considered only women for which both first post-contrast MRI T1 and MRI T2 exams were available (Figure 1). MRI T1 exams allow us to detect tumor characteristics at initial diagnosis regardless of any type of therapy, while MRI T2 exams show tumor properties that are already informative of the therapy efficacy [5,6,7,8,9]. With this kind of analysis, the current work wanted to respond to the request for designing models based on image processing with the aim to predict RFS or BCR “early on” implicitly, including information about the efficacy of the therapy. The second selection criterion refers to the temporal interval to the occurring of the recurrence event. As highlighted by some information from the I-SPY1 TRIAL database, available online [33], a binary Recurrence-Free Survival indicator (RFSi) can be defined depending on whether the patients experienced recurrence or not from the date of chemotherapy initiation, which is commonly chosen as a starting point to calculate RFS time (Figure 1) [13,34]. The original I-SPY1 TRIAL dataset provides patients for which recurrence occurred within a maximum period of about six years from chemotherapy initiation and recurrence-free patients for which the last follow-up varied from one year to more than six years from chemotherapy initiation. In this study, in order to split patients into a control group (that had not shown recurrence and were hence recurrence-free, RFSi) and an experimental group (that showed recurrence and were hence non-recurrence-free, non-RFSi) and, at the same time, to involve the highest number of patients, we considered only those patients with at least three years follow-up from the beginning of NACT or an event within that timeframe. A similar rationale was applied to patients of the BREAST-MRI-NACT-Pilot database to define a control group and an experimental group. Therefore, a set of 121 patients and a set of 37 patients were identified from the I-SPY1 TRIAL and the BREAST-MRI-NACT-Pilot databases, respectively. Because the databases were composed of high-resolution images [1,31], we did not apply other exclusion criteria, e.g., based on low image quality and inflammatory breast cancers. Table 1 shows the clinical details, i.e., average age and receptor status, of the identified patients, distinguishing them according to class (non-RFSi vs. RFSi).

As interesting observation is that the high rate of HER2+ patients that showed recurrence (non-RFSi in Table 1) could be related to a non-association between taxane and trastuzumab during chemotherapy. Indeed, the patients were enrolled for the studies related to the construction of the I-SPY1 TRIAL and the BREAST-MRI-NACT-Pilot databases between 2002 and 2006 and between 1995 and 2002, respectively, while neoadjuvant trastuzumab was not used as standard therapy until 2005 [15].

Two subsets of patients were then taken into account. The first subset, hereafter referred to as *fine-tuning dataset*, involved 96 patients from the I-SPY1 TRIAL database, out of which 26 patients showed recurrence (non-RFSi) and 70 did not (RFSi). This subset was used to find the most stable set of deep convolutional neural network features. The second subset, made up of 62 patients out of which 25 patients (8 non-RFSi and 17 RFSi) from the I-SPY1 TRIAL dataset and 37 patients (9 non-RFSi and 28 RFSi) from the BREAST-MRI-NACT-Pilot dataset, was used as an *independent test* to validate the proposed model on previously unseen patients.

### 2.2. Data Pre-Processing

After the MRI scans were successfully downloaded, the slices with the largest tumor area of the first post-contrast MRI T1 and T2 exams were separately considered. For patients belonging to the *fine-tuning dataset* and patients of the *independent test* from BREAST-MRI-NACT-Pilot database, tumor segmentations related to the selected exams were already available in the online dataset and were automatically generated by thresholding semi-quantitative pharmacokinetic parameters, peak enhancement, and signal enhancement ratio. For patients belonging to the *independent test* from the I-SPY1 TRIAL database, instead, tumor segmentations were manually generated by one radiologist of our Institute with 20 years of experience in breast cancer diagnosis. An automatic procedure was then implemented to localize the center of the tumor mass and to extract, from the chosen MRI exam, a Region Of Interest (ROI). The size of the ROI was imposed equal to the Largest Diameter (LD) of the tumor, whose value was reported in a file available online as part of the databases [33]. Overall, the *independent test* was composed of either automatically or manually segmented images. Finally, two ROIs, respectively related to the first post-contrast MRI T1 and T2 exams, were used for each patient as inputs of the neural network for feature extraction. All the ROIs were resized as patches of 227 × 227 pixels using bicubic interpolation, because the network used as the feature extractor requires input images with dimensions of 227 × 227 pixels.

### 2.3. A Hybrid Machine Learning-Deep Learning Approach

The approach proposed in this paper can be considered as a hybrid machine learning-deep learning method given that we used a deep learning architecture, namely, the pre-trained Convolutional Neural Network (CNN), AlexNET [35], as a feature extractor, combined with a classical machine learning algorithm, namely, a Support Vector Machine (SVM) with linear kernel [36], as classifier. The proposed model was evaluated on the prediction of three-year BCR that was formulated as a binary discrimination problem, i.e., RFSi vs. non-RFSi. The performance of the prediction model was assessed in terms of accuracy, sensitivity, specificity, and the Area Under the receiver operating Curve (AUC).

Accuracy indicates the rate of correct classification between recurrent-free and non-recurrent-free cases. Sensitivity and specificity measure the proportion of non-recurrence-free cases (non-RFSi) and the proportion of recurrence-free cases (RFSi) that are correctly identified, respectively. AUC, instead, evaluates the ability of the classifier to distinguish between the two classes (RFSi vs. non-RFSi) with values ranging from 0.50, meaning random guessing, to 1, meaning perfect separability.

**Figure 2:**
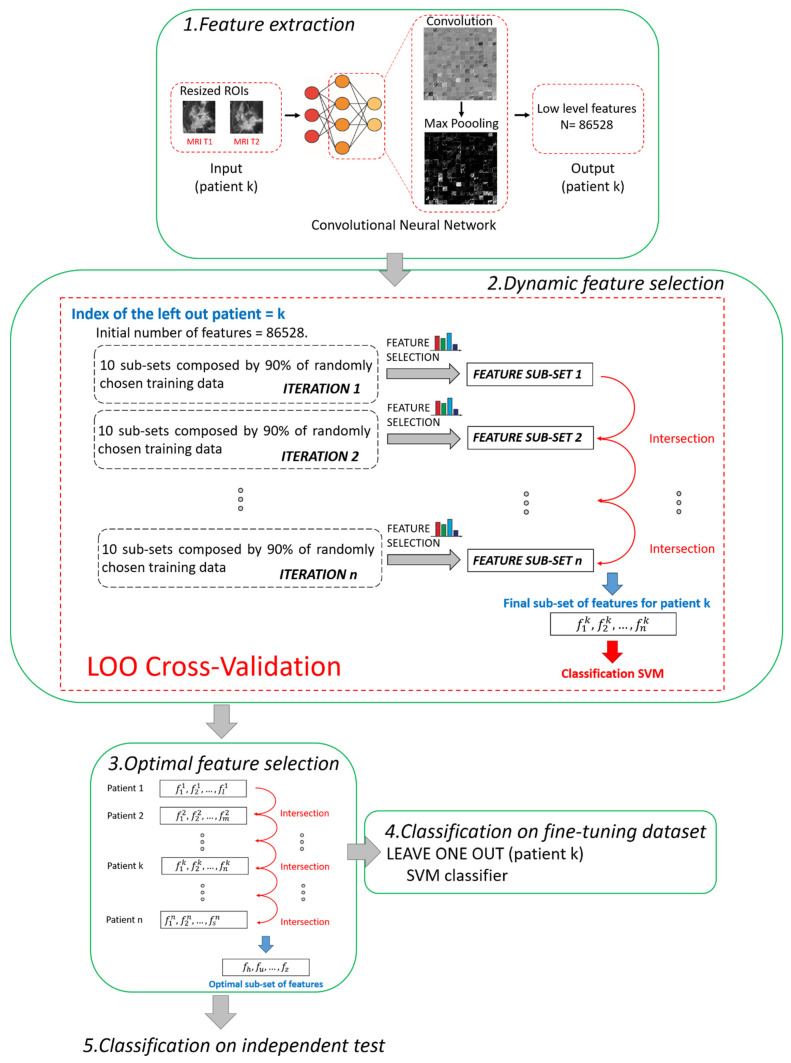
Pipeline of the proposed hybrid machine learning-deep learning method. Five main steps can be distinguished: 1. Feature extraction, 2. Dynamic feature selection, 3. Optimal feature selection, 4. Classification on fine-tuning dataset, 5. Classification on independent test.

The main steps of the proposed method are summarized and depicted in Figure 2. All the steps were performed using MATLAB R2019a (MathWorks, Inc., Natick, MA, USA) software.

#### 2.3.1. Step 1. Feature Extraction

A pre-trained deep learning architecture based on CNN, called AlexNET [35], was adopted to automatically extract features from the two ROIs (one from MRI T1 and one from MRI T2) associated to each patient. The network AlexNET is composed of eight main layers: five convolutional layers and three fully connected layers. ReLU Nonlinearities and overlapping pooling layers are interlaced with the main ones so that the network counts 25 layers in total [35]. The choice of a pre-trained network as a feature extractor was inspired from the latest successful implementation of the so-called *knowledge learning* or *transfer learning* across multiple research or real-world applicative domains [23,37,38]. Basically, the core idea of transfer learning is to overcome the constraints of many machine learning algorithms that work well only when the training and the test samples are derived from the same distribution or the same feature space: collecting new training data is required when distribution changes. Thanks to transfer learning, instead, it is possible to exploit a huge amount of training samples of a certain field of application (pre-trained networks) and transfer the acquired knowledge on unseen data drawn from a new feature space or distribution [21]. For example, AlexNET has been previously trained on more than 15 million natural (non-medical) images labelled in 22 thousand classes [35]. Transfer leaning is here applied to MRI images. Two other advantages can be highlighted. First, a huge number of features can be automatically created without human bias (*unperceivable-to-human* features), and complex non-linear relationships among features can be caught. Second, the use of a pre-trained network may avoid time consumption related to the retraining of the network and even the risk of overlearning due to the reduced number of available images in real-word applications, as in our study.

In this work, low-level features, i.e., details of images such as lines, edges, or blobs, derived from one of the starting convolutions within the network architecture [39], were considered to fulfil the prediction task. We preferred to use low-level features instead of high-level features related to the last layers of the network because low-level features pay more attention to the local structure of the image. On the contrary, high-level features tend to capture more global cues, such as shapes and objects in their entirety, thus losing or neglecting potentially useful information from local structures. Moreover, the features were extracted after pooling because it has been demonstrated that, by pooling, the derived features show higher invariance to truncation, occlusion, and translation [40]. In detail, the features were extracted from the 9th layer of the network, named the *pool2 layer*, as it corresponds to the second pooling layer (Max Pooling in phase 1 of Figure 2) after the second convolutional layer of the network (Convolution in phase 1 of Figure 2). The *pool2 layer* had an output with dimensions of 13 × 13 × 256 that was flattening to a single 43264-length vector. Because, for each ROI, the total number of extracted features was 43264, each patient (represented by two ROIs) had 86528 features counted (43264 for the ROI from MRI T1 and 43264 for the ROI from MRI T2).

#### 2.3.2. Step 2. Dynamic Feature Selection

The *fine-tuning dataset* was used at this step. Because we had to deal with a high-dimensional dataset, we combined two different kinds of feature selection methods, filter and embedded strategies [41], to address the issue of high dimensionality.

In this work, the non-parametric Wilcoxon–Mann–Whitney test [42] was used as the filter method: the medians of the distributions related to the two classes (RFSi vs. non-RFSi) were compared and verified as to whether they were equal. A *p*-value equal to 0.001 was set as the cut-off to indicate a statistically significative difference between pairs of analyzed distributions.

Moreover, we used Random Forest (RF) [43] as an embedded criterion. It is based on a tree strategy that evaluates the so-called Gini impurity to identify the most important features. The configuration of RF counted 100 trees.

The two feature selection methods were employed for a *dynamic* feature selection, based on a Leave-One-patient-Out (LOO) cross validation procedure. The term *dynamic* means that the subset of features, selected leaving one patient out, differs from one patient to another. At each step of LOO cross-validation, a hierarchical training splitting procedure was preferred to a simple training splitting procedure in order to make the feature selection procedure more robust to changes in training data [21].

The hierarchical training splitting procedure can be defined as an iterative process. At each iteration, 10 subsets of training data, each one composed of 90% of the training samples (see phase 2 in Figure 2), were randomly chosen. For each of the subsets, the filter selection technique, followed by the embedded criterion, was applied. The features selected for each subset of data were joined together in a single set. In this way, at the *i*th iteration corresponded the *i*th sub-set of features. The sub-sets of features obtained from the following iterations were intersected among each other. The basic idea was to execute iterations to reduce the number of features. By increasing iterations, the number of features tended to stabilize, thus reducing noise and redundancy among the selected features. The iterative procedure continued until the number of features discarded from two subsequent iterations was less than 10 features for 95% of the patients. As a result of the implementation, the total number of iterations was empirically estimated as *n =* 20. The final selected features were used to build an SVM classifier.

#### 2.3.3. Step 3. Optimal Feature Selection

According to research works that tackle the issue of the stability of the selected features [44,45], feature selection techniques can usually be sensitive to variations of the training subsamples. The poor stability of features could negatively influence the performance of the model. The dynamic feature selection implemented in Step 2 partially solved the issue by identifying a different subset of features in correspondence to each patient. However, we wanted to detect a unique set of the most stable features, as small as possible and valid for any patient, thus increasing the strength of the model and obtaining a more accurate classification. Hence, an Optimal Subset of Features (OSF) was created with the most stable features, namely, the features that were present in each subset of features (one for each patient) found after applying Step 2. In other words, the subset of optimal features was obtained by intersecting all the subsets of features patient by patient (see phase 3 in Figure 2).

#### 2.3.4. Step 4. Classification on Fine-Tuning Dataset

The OSF identified at Step 3 was used to build an SVM classifier on patients belonging to the *fine-tuning dataset*. Moreover, another SVM classifier was implemented by increasing the number of features thanks to the addition of four clinical features: age, ER, PgR, and HER2+ (see Table 1). Classification model training, as well as its performance evaluation, were performed by a leave-one-patient-out cross-validation procedure.

#### 2.3.5. Step 5. Classification on Independent Test

As the last step, we evaluated the robustness of the presented method on the *independent test*. The *fine-tuning dataset* was used as the training set to train two diverse SVM classifiers; the first model exploited the identified OSF, the other one employed the OSF with the addition of the four above mentioned clinical features.

## 3. Results

A threefold evaluation of the proposed model was performed. More specifically, the performance of the model was assessed on the *fine-tuning dataset* by using features extracted by means of the dynamic feature selection (Step 2) and the optimal feature selection (Steps 3–4), respectively. The key role of the *fine-tuning dataset* was to find the most stable features. Next, the model with optimal features was validated on an *independent test* whose patients were not involved in the feature selection process (Step 5).

### 3.1. Predictive Contribution of the Features Extracted Solely from MRI T1 Exams

Firstly, we investigated the predictive contribution of the features selected solely from the pre-treatment exams, that is, by considering only the features extracted from ROIs related to MRI T1 exams.

**Figure 3:**
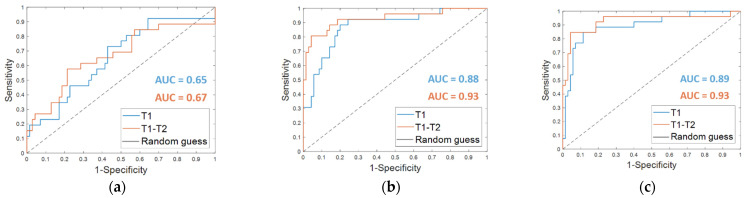
ROC curves for breast cancer recurrence prediction models on the *fine-tuning dataset* (**a**) using the Dynamically Selected Features, (**b**) the Optimal Subset of Features alone and (**c**) combined with the clinical features, respectively. The AUC values are also highlighted.

From the classification with the Dynamically Selected Feature (DSF) on the *fine-tuning dataset*, an accuracy of 67.7%, a sensitivity of 38.5%, a specificity of 78.6%, and an AUC value of 0.65 were obtained (Table 2, DSF T1). The corresponding ROC curve is plotted in Figure 3a (T1 in blue).

The OSF found for timepoint T1 (OSF T1) counted 15 features, of which 11 were in common with the OSF T1–T2, i.e., the optimal subset of CNN-extracted features obtained by implementing Step 4 of the proposed method when both MRI T1 and MRI T2 exams were analyzed (see the following subparagraph). Table 2 summarizes the performances reached on the *fine-tuning dataset* by using the OSF T1, which are an accuracy of 82.3%, a sensitivity of 57.7%, and a specificity of 91.4%. The results were improved by adding the clinical features (OSF + clinical T1), obtaining an accuracy of 87.5%, a sensitivity of 69.2%, and a specificity of 94.3%. The AUC value passed from 0.88 to 0.89. The corresponding ROC curves are represented in Figure 3b,c (T1 in blue), respectively.

**Figure 4:**
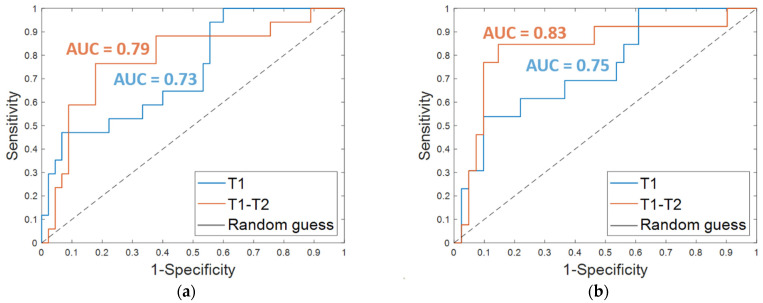
ROC curves for breast cancer recurrence prediction models on the *independent test* (**a**) using the Optimal Subset of Features alone and (**b**) combined with the clinical features, respectively. The AUC values are also highlighted.

The results obtained on the *independent test* are listed in Table 3. An accuracy of 80.7%, a sensitivity of 47.1%, a specificity of 93.3%, and an AUC value of 0.73 were obtained by using OSF T1. The corresponding ROC curve is plotted in Figure 4a (T1 in blue). The addition of clinical features led to an increase of the sensitivity (53.9%) and the AUC value (0.75, Figure 4b, T1 in blue) and to a slight decrease of the specificity (90.2%), thus returning a similar accuracy (80.3%).

### 3.2. Breast Cancer Recurrence Prediction Using Features Extracted from MRI T1 and T2 Exams

Starting from the initial 86528 features, the proposed dynamic feature selection procedure returned more or less than 100 features for each of the patients of the *fine-tuning dataset*. The SVM classifier built using these DSF features returned an accuracy of 72.9%, a sensitivity of 57.7%, a specificity of 78.6 % (reported in Table 2, DSF. T1–T2), and an AUC value of 0.67 (Figure 3a, T1–T2, in orange).

The OSF identified by implementing *Step 4* of the hybrid machine learning-deep learning method counted 18 features overall: 13 features for the pre-treatment exams, MRI T1, and 5 for the early-treatment exams, MRI T2 (OSF T1–T2).

When OSF T1–T2 was used (Table 2, OSF T1–T2), the classification performance on the *fine-tuning dataset* reached an accuracy of 87.5%, a sensitivity of 80.8%, a specificity of 90.0 %, and an AUC value of 0.93. Fusing the OSF T1–T2 with the clinical variables (Table 2, OSF + clinical T1–T2) resulted in higher values of accuracy (91.7%) and specificity (95.7%). The sensitivity as well as the AUC value remained unvaried. The related ROC curves are represented in Figure 3b,c (T1–T2 in orange), respectively.

Promising results were reached on the *independent test*; an accuracy of 80.7%, a sensitivity of 76.5%, and a specificity of 82.2% were achieved using the OSF T1–T2 alone (Table 3, OSF T1–T2). When the features of the OSF T1–T2 were combined with the four clinical features (Table 3, OSF + clinical T1–T2), an accuracy of 85.2%, a sensitivity of 84.6%, and a specificity of 85.4% were reached. Additionally, the AUC value was improved with the addition of the clinical variables. Indeed, using only the OSF T1–T2 resulted in an AUC of 0.79 (Figure 4a, T1–T2 in orange), whereas integrating the model with the clinical variables resulted in an AUC of 0.83 (Figure 4b, T1–T2 in orange).

Finally, the results achieved on the *independent test* by considering the features extracted and selected from the pre-treatment exams alone, and thus referred to tumor mass at initial diagnosis regardless of any therapy, were quite promising. The addition of information extracted from the early-treatment exams and hence related to the tumor mass after the first cycle of treatment allowed us to globally enhance performances. In detail, when only the OSF T1 was used, a higher specificity was reached, but at the expense of sensitivity, which was quite low. By using OSF T1–T2, more balanced evaluation metrics and a greater increment of sensitivity were achieved (refer to the last two columns of Table 3). An improvement of the ability of the classifier to discern between recurrence-free and non-recurrence-free cases, expressed by AUC, was also achieved, and the maximum value was reached after the addition of the clinical variables.

### 3.3. Feature Visualization

**Figure 5:**
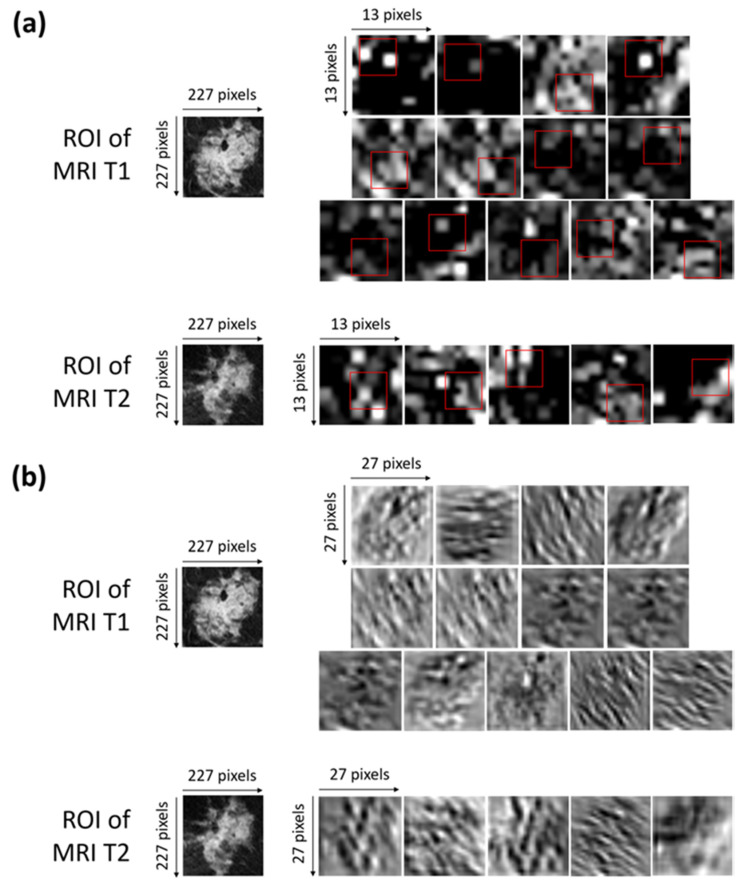
(**a**) Activation maps of the ROIs of both of the MRI T1 and MRI T2 exams from which the selected optimal features were extracted. The red squares outline, with more precision, the areas belonging to such features. Each ROI has dimensions of 227 × 227 pixels. Each activation map has original dimensions of 13 × 13 pixels and has been resized to the dimensions of the ROIs for a better visualization. (**b**) Convolutional maps related to the activation maps of the ROIs of both the MRI T1 and MRI T2 exams from which the selected optimal features were extracted. Each ROIs has dimensions of 227 × 227 pixels. Each convolutional map has original dimensions of 27 × 27 pixels and has been resized to the dimensions of the ROIs for better visualization.

Neural networks have made great advances and continue to have great success, though the so-called *explainability* of their internal structures and the consequent mechanisms of transfer of information are still open problems [46]. In other words, a neural network can be considered as a ‘black box’ because the transformation of the input into the output is based on an approximation of a mathematical function of which the network cannot give us any insights as to its form [47]. There is not a trivial link between the weights of the network and the approximation of the function. Indeed, a great number of parameters, usually called hyperparameters, influence the function being approximated. Some experts are addressing the ‘black box’ problem as the main focus of their research, with an attempt to interpret the roles of the inner layers [48,49]. Concerning the interpretation of the inner layer, in our work, we have represented, in Figure 5a,b, the activation and convolutional maps of the selected optimal characteristics.

More precisely, Figure 5a shows the activation maps of the ROIs related to both the MRI T1 and MRI T2 exams from which the selected optimal features were extracted. The red squares outline, with more precision, the areas belonging to such features. The activation maps (Figure 5a) were obtained as outputs of the Max Pooling layer (Max Pooling in phase 1 of Figure 2) after sequentially applying the Rectified Linear Units (ReLU) activation function, normalization, and max pooling to the output of the convolutional layer [35].

The interpretation of the extracted features is not so trivial. As a general consideration, low-level features that represent local structures, such as edges or blobs, were extracted from the available ROIs. By observing the areas to which the optimal features belong and roughly comparing them with the corresponding areas in the original ROIs, we can say that the largest part of the features comprising the OSF T1–T2 are related to edge cues or the central part of the tumor masses within the ROIs.

Figure 5b shows the convolutional maps related to the activation maps of the ROIs of both the MRI T1 and MRI T2 exams from which the selected optimal features were extracted (output of the convolutional layer represented in phase 1 of Figure 2). The visualization of the convolutional maps allows us to understand the operations applied to finally obtain the convolutional maps, which are then transformed into activation maps. Basically, each convolutional map shows what kind of lines or details were extrapolated from the corresponding original image.

## 4. Discussion

In the state-of-the-art, there are a number of works that make use of predictive models or radiomic analysis to identify and characterize breast neoplasms and the state of lymph node involvement [50,51,52,53,54,55,56].

In this work, we have developed a hybrid machine learning-deep learning approach to give an early prediction of three-year BCR for patients undergoing NACT, using DCE-MRI from I-SPY1-TRIAL and BREAST-MRI-NACT-Pilot databases. Basically, we leveraged the power of a pre-trained CNN to automatically extract meaningful representations from raw MRI exams combined with a standard SVM classifier. To supply an early prediction of BCR, only the MRI exams acquired at the first two visits (pre-treatment, MRI T1; early-treatment, MRI T2) were analyzed. As far as we can tell, this is the first work that exploits CNN-extracted features for an early prediction of recurrence for BC patients undergoing NACT.

Recurrence prediction for BC patients, including patients whose cases are referred to in the I-SPY1 TRIAL and BREAST-MRI-NACT-Pilot databases, has been usually handled as recurrence-free-survival prediction, for which radiomic analysis using regression models with handcrafted features have represented the most common choice [16,17,18,32]. The regression models have been developed for the so-called survival analysis [57,58], which is based on the inquiry of the association between one or more predictor variable candidates and the RFS considered as survival time. Their performances are usually evaluated in terms of C-statistic, which is a metric equivalent to AUC [59]. In this study, instead, we formulated the breast cancer recurrence prediction as a classification task to distinguish patients that showed recurrence from those that did not. Women with at least three years follow-up from the beginning of NACT or an event within that timeframe were considered in accordance with data availability. The recurrence-free survival indicator (RFSi) has been considered to distinguish patients who experienced recurrence (non-recurrence-free) and patients who did not experienced recurrence (recurrence-free), respectively (non-RFSi vs. RFSi). Moreover, the regression models often make use of clinical variables as well as radiomic features designed by data scientists [17,18,19]. To give some examples, Hylton et al. [16] were pioneers in demonstrating that the Functional Tumor Volume (FTV) computed from the DCE-MRI of 163 patients was predictive of recurrence-free survival. At univariate analysis, mean C-statistic values of 0.67 and 0.64 were reached for FTV at time T2 and FTV at time T4, respectively. At multivariate analysis, the model including FTV at time T2 and clinical variables had the strongest predictive performance, showing a mean C-statistic value of 0.72. Janahi et al. [17,18] derived four features related to the texture of voxel-level changes in tumor kinetic response between registered images of MRI T1 and T2 of a set of 106 patients. Then, they designed a regression model by combining the extracted kinetic features with some clinical features and reached a mean C-statistic value of 0.76.

More recently, the BCR prediction interpreted as a classification task (RFSi vs. non-RFSi) was tackled by Drukker et al. [11]. The authors used eight kinetic features to build a classifier based on long-short term memory networks. In order to predict BCR at two years post-surgery on 157 patients, (132 non-RFSi; 24 RFSi) they exploited MRI scans from T1 to T4 time points. Conversely to our study, where we considered only women for which both MRI T1 and MRI T2 exams were available, Drukker et al. involved in their study all patients with at least one MRI scan from T1 to T4. In this way, a different number of MRI exams were analyzed at each treatment time point. They also showed the results related to the subgroup of patients by analyzing the exams related to the two first visits, i.e., MRI T1 and/or T2; an AUC value of 0.75 was reached.

All the research works previously mentioned, whether they were based on regression or classification models, have obtained promising results to predict RFS and BCR, respectively. However, they are crucially influenced by manual feature extraction. Beyond requiring a huge human effort, the reliability of handcrafted features depend upon human expertise in the field. Additionally, they can lose generalizability because they involve parameters manually set at varying the image of application: we can refer, for example, to thresholds related to image gradients in texture-based features.

Within this emerging scenario, we investigated the prediction of BCR from a new perspective. The image-coding developed in this study allows overcoming the limitations of handcrafted features, taking advantages of the application of a pre-trained CNN that is able to learn the knowledge acquired on an enormous number of images belonging to thousands of categories during the pre-training phase and transfer it onto new unseen images of interest, such as medical images. In addition, using a pre-trained network allows us to reduce time consumption with respect to training a network on specific data (customized networks). An advantage is also the improvement of either generalizability or accuracy with respect to custom-built networks that can be more prone to overfitting due to the small amount of available data of the real-word applications, as can be seen in our study. Additional novel aspects of the proposed approach include the investigation of feature stability through the designing of a model with an optimal subset of features, which were found as the most stable CNN extracted features (18 features, of which 13 were MRI T1 exams and 5 were MRI T2 exams, respectively).The identification of the most stable features is a very valuable aspect to take into account because large datasets of features can be affected by redundancy and sampling perturbations [44,45]. The validation of an independent test containing either manually or automatically segmented images as well as the fusion of deep learning with clinical features to improve recurrence prediction have been investigated. The latter fusion led to the best performance on the independent test, reaching an AUC value of 0.83. The predictive contribution related to features extracted and selected solely from the pre-treatment exam was also investigated; AUC values of 0.73 and 0.75 were achieved with the CNN features alone or combined with the clinical ones. In Section 3 (Results), we summarized the results that we considered as most significant for the purpose of the study. For example, we did not report the results achieved by analyzing the early-treatment exams alone and the only clinical variables. However, in our preliminary analysis, the optimal selected features for timepoint T2 counted eight features, of which five were in common with the OSF T1–T2. The performances of the early-treatment exams alone did not appear significantly better than those achieved by analyzing the pre-treatment exams alone (AUC value of 0.72 for the independent test). We also assessed the discriminative power of the only clinical features; when joined together to design an SVM classifier, the clinical variables alone (ER, PgR, HER2+, and age) did not show a good predictive performance. In this work, our challenge was to investigate the role of automatically extracted features by a transfer learning-based algorithm. In particular, we made use of features extracted from the first layers of the network. They are low-level features, i.e., they dissect information regarding the local structure of the row images, with the ability to catch information and complex non-linear relations that can be neglected by experts during the process of feature engineering. From the promising experimental results achieved, the new evidence that we found is that these kinds of feature, which describe patterns and details that humans cannot perceive, have been revealed to be powerful and can give an early prediction of recurrence for BC patients undergoing NACT. Compared with the other approaches, the analysis of the images based on the proposed feature extraction and selection resulted as very promising. This means these features give added value to fulfill the BCR prediction task. Under such considerations, this work represents a primary effort to open the path to a deeper and wider investigation of CNN extracted features within this framework. After further investigations, validations, and extensions, we believe that joining these automatically extracted features with some existing and well-defined radiomic features and also clinical features, such as histopathological or genomic features, could have a relevant impact on clinical practice, with a greater improvement of the predictive performances for BCR.

As a crucial point of discussion, we can emphasize that the ease of implementation and interpretation could make the proposed method a powerful tool in support of decision-making capabilities in the current clinical workflow, with the possibility to make changes and improvements of the therapy pathway in its early stages and hence before surgery. It is made possible by automatically analyzing and combining tumor characteristics prior to any therapy (from pre-treatment exams) with those detected after its early stage (from early-treatment exams), which can arguably give us an indication of the effectiveness of therapy. In this way, the model, which has been developed to give an early prediction of recurrence, implicitly includes information about the efficacy of the therapy. However, a limitation of this study is that the dataset was relatively small in size. Moreover, a drop of accuracy by passing from the fine-tuning dataset to the independent test was observed. The independent test can be considered as a heterogeneous dataset because it contained, on the one side, manually segmented images from the same database to whom the patients of the fine-tuning dataset belonged (I-SPY1 TRIAL database), and, on the other side, automatically segmented images from another database (BREAST-MRI-NACT-Pilot database). Anyway, the reached results are lower than those achieved on the fine-tuning dataset but constitute a promising basis for a more extensive validation. The validation and extension of the proposed model on a larger dataset of patients who are afferent to our Institute and multicenter research projects will be handled in the future, also supported by a specific study of the robustness and generalizability of the model.

After large-scale validation, the envisioned innovation potential of the proposed tool could be applied on multiple neoadjuvant therapy schemes, and the probability of recurrence related to every specific therapy pathway could be computed. Thanks to this information, clinicians could be able to choose the therapy framework to which a lower probability of recurrence is associated.

According to ASCO guidelines [15], patients with node-positive or high-risk node-negative, HER2-positive disease should be offered neoadjuvant therapy with an anthracycline and taxane or non-anthracycline-based regimen in combination with trastuzumab. Pertuzumab may be used with trastuzumab in the neoadjuvant setting, while in patients with triple negative breast cancer (TNBC), who have clinically node-positive and/or at least T1c disease, an anthracycline and taxane-containing regimen is the most appropriate treatment. Carboplatin may be considered as part of a neoadjuvant regimen in patients with TNBC to increase the likelihood of therapy efficacy.

As explanatory example, a percentage of 83.7% of the total number of non-recurrence-free (non-RFSi) patients involved in this study (43, see Table 1), did not achieved pathological complete response. If this tool had been available at the time of the public databases construction, it would have better driven therapeutic decisions, e.g., by adding trastuzumab to the therapy finalized for HER2+ patients.

Finally, as extensively underlined in the paper, we focused on the early prediction of breast cancer recurrence using pre-treatment and early-treatment MRI exams. Inter-regimen and pre-surgery MRI scans were left out of our study because we were interested on early prediction of recurrence. An interesting future analysis step could include the analysis of inter-regimen and pre-surgery MRI exams to combine recurrence prediction with partial and complete pathological response to the therapy, respectively.

## 5. Conclusions

Predictive models for an early prediction of recurrence-free survival for breast cancer patients undergoing NACT may represent a valuable support tool for radiologists and oncologists to identify the most suitable treatment planning for individual patient scenarios. The high value of this kind of tool exists in the possibility to modify and/or intensify the chemotherapeutic regimen after a first assessment of therapy efficacy through MRI analysis, in order to obtain the best therapeutic response before surgery.

Nevertheless, because the early prediction of breast cancer recurrence is a quite challenging task, only a few methods have been investigated in the state-of-the-art. In this paper, we addressed the task under a new outlook based on the exploitation of the well-known transfer learning technique using breast MRI scans from the I-SPY1 TRIAL and the BREAST-MRI-NACT public datasets. More specifically, we developed a framework pairing a pre-trained deep learning architecture with a standard machine learning method to give an early prediction of BCR within three years from the NACT beginning. With this aim, only pre-treatment and early-treatment MRI examinations acquired after the first post-contrast injection were analyzed. The issue of feature stability was investigated, and an optimal subset of features was found. Both the robustness and versatility of the proposed method have been demonstrated through its evaluation on an independent test involving either manually or automatically segmented images, achieving a good prediction performance. Combining the model with clinical features improved the performance. As a further extension of the study, the low-level unperceivable-to-human features extracted by the network could be combined with a wider number of clinical features of different kinds, such as features related to histopathology or gene expression profiling. Future work will concern the validation of the proposed method on larger datasets and its application on a wider range of neoadjuvant therapy frameworks whose efficacy could be evaluated by the predicted probability of recurrence. Therapy pathways related to a lower probability of recurrence could be preferred by medical experts.

Finally, a powerful envisioned automatic platform could be designed to combine the early prediction of recurrence probability with the early prediction of the pathological complete response. The key idea is that, given a patient undergoing a neoadjuvant chemotherapy scheme, a first evaluation of the therapy efficacy in terms of pCR prediction can be performed after the early stage of the therapy itself. Such an evaluation could be strengthened or weakened by the prediction of the recurrence probability provided by the predictive transfer learning approach proposed in the current study.

## Figures and Tables

**Table 1:** Clinical specifications of the patients involved in the study. The acronym non-RFSi indicates non-recurrence-free patients and hence those patients who showed recurrence. The acronym RFSi stands for recurrence-free patients and hence those patients who did not show recurrence.

Characteristic	Non-RFSi	RFSi
Number (*n* = 158)	43	115
Average Age (years)	47.21 ± 8.67	48.86 ± 8.96
Receptor Status		
ER Positive	12	66
PgR Positive	14	53
HER2 Positive	17	26

**Table 2:** Summary of the performances of the breast cancer recurrence prediction models on the *fine-tuning dataset* in terms of accuracy, sensitivity, and specificity, using the Dynamically Selected Features (DSF), the Optimal Subset of Features (OSF), also combined with the clinical features (OSF + clinical), respectively. The features were selected for the exams at timepoint T1 alone or at timepoints T1–T2 simultaneously (original model). The numbers of features selected for OSF and OSF + clinical are also highlighted. The numbers of DSF features are omitted because they vary from one patient to another.

Fine-Tuning Dataset						
Performance Metric	DSF		OSF		OSF + Clinical	
	T1	T1–T2	T1	T1–T2	T1	T1–T2
***N*. features**	-	-	15	13 + 5	15 + 4	13 + 5 + 4
**Accuracy**	67.7%	72.9%	82.3%	87.5%	87.5%	91.7%
**Sensitivity**	38.5%	57.7%	57.7%	80.8%	69.2%	80.8%
**Specificity**	78.6%	78.6%	91.4%	90.0%	94.3%	95.7%

**Table 3:** Summary of the performances of the breast cancer recurrence prediction models on the *independent test* in terms of accuracy, sensitivity, and specificity, using the Optimal Subset of Features (OSF) also combined with the clinical features (OSF + clinical), respectively. The features were selected for the exams at timepoint T1 alone or at timepoints T1–T2 simultaneously (original model). The number of features selected for OSF and OSF+ clinical are also highlighted.

Independent Test				
Performance Metric	OSF		OSF + Clinical	
	T1	T1–T2	T1	T1–T2
***N*. features**	15	13 + 5	15 + 4	13 + 5 + 4
**Accuracy**	80.7%	80.7%	80.3%	85.2%
**Sensitivity**	47.1%	76.5%	53.9%	84.6%
**Specificity**	93.3%	82.2%	90.2%	85.4%

## Data Availability

Data images refer to the public databases that are part of The Cancer Imaging Archive (TCIA) at the following link: https://wiki.cancerimagingarchive.net (accessed on 1 August 2020).

## References

[1] Hylton N.M., Blume J.D., Bernreuter W.K., Pisano E.D., Rosen M.A., Morris E.A., Weatherall P.T., Lehman C.D., Newstead G.M., Polin S. (2012). Locally advanced breast cancer: MR imaging for prediction of response to neoadjuvant chemotherapy–Results from ACRIN 6657/I-SPY TRIAL. Radiology.

[2] Song S.E., Seo B.K., Cho K.R., Woo O.H., Son G.S., Kim C., Cho S.B., Kwon S.-S. (2015). Computer-aided detection (CAD) system for breast MRI in assessment of local tumor extent, nodal status, and multifocality of invasive breast cancers: Preliminary study. Cancer Imaging.

[3] Lee M.C., González S.J., Lin H., Zhao X., Kiluk J.V., Laronga C., Mooney B. (2015). Prospective Trial of Breast MRI Versus 2D and 3D Ultrasound for Evaluation of Response to Neoadjuvant Chemotherapy. Ann. Surg. Oncol..

[4] Krug D., Baumann R., Budach W., Dunst J., Feyer P., Fietkau R., Haase W., Harms W., Hehr T., Piroth M.D. (2018). Neoadjuvant chemotherapy for breast cancer—background for the indication of locoregional treatment. Strahlenther. Onkol..

[5] Rousseau C., Devillers A., Sagan C., Ferrer L., Bridji B., Campion L., Ricaud M., Bourbouloux E., Doutriaux I., Clouet M. (2006). Monitoring of early response to neoadjuvant chemotherapy in stage II and III breast cancer by [18F]fluorodeoxyglucose positron emission tomography. J. Clin. Oncol..

[6] Marinovich M.L., Sardanelli F., Ciatto S., Mamounas E., Brennan M., Macaskill P., Irwig L., von Minckwitz G., Houssami N. (2012). Early prediction of pathologic response to neoadjuvant therapy in breast cancer: Systematic review of the accuracy of MRI. Breast.

[7] Khairalseed M., Javed K., Jashkaran G., Kim J.W., Parker K.J., Hoyt K. (2019). Monitoring early breast cancer response to neoadjuvant therapy using H-scan ultrasound imaging: Preliminary preclinical results. J. Ultrasound Med..

[8] Tan W., Yang M., Yang H., Zhou F., Shen W. (2018). Predicting the response to neoadjuvant therapy for early-stage breast cancer: Tumor-, blood-, and imaging-related biomarkers. Cancer Manag. Res..

[9] Sharma U., Danishad K.K.A., Seenu V., Jagannathan N.R. (2009). Longitudinal study of the assessment by MRI and diffusion-weighted imaging of tumor response in patients with locally advanced breast cancer undergoing neoadjuvant chemotherapy. NMR Biomed..

[10] Drukker K., Li H., Antropova N., Edwards A., Papaioannou J., Giger M.L. (2018). Most-enhancing tumor volume by MRI radiomics predicts recurrence-free survival ‘early on’ in neoadjuvant treatment of breast cancer. Cancer Imaging.

[11] Drukker K., Edwards A., Papaioannou J., Giger M. Long short-term memory networks predict breast cancer recurrence in analysis of consecutive MRIs acquired during the course of neoadjuvant chemotherapy. Proceedings of the Medical Imaging 2020: Computer-Aided Diagnosis.

[12] Relapse-Free-Survival Definition. https://www.cancer.gov/publications/dictionaries/cancer-terms/def/relapse-free-survival.

[13] Hudis C.A., Barlow W.E., Costantino J.P., Gray R.J., Pritchard K.I., Chapman J.-A.W., Sparano J.A., Hunsberger S., Enos R.A., Gelber R.D. (2007). Proposal for standardized definitions for efficacy end points in adjuvant breast cancer trials: The STEEP system. J. Clin. Oncol..

[14] Ding Y., Ding K., Yang H., He X., Mo W., Ding X. (2020). Does dose-dense neoadjuvant chemotherapy have clinically significant prognostic value in breast cancer?: A meta-analysis of 3724 patients. PLoS ONE.

[15] Korde L.A., Somerfield M.R., Carey L.A., Crews J.R., Denduluri N., Hwang E.S., Khan S.A., Loibl S., Morris E.A., Perez A. (2021). Neoadjuvant Chemotherapy, Endocrine Therapy, and Targeted Therapy for Breast Cancer: ASCO Guideline. J. Clin. Oncol..

[16] Hylton N.M., Gatsonis C.A., Rosen M.A., Lehman C.D., Newitt D.C., Partridge S.C., Bernreuter W.K., Pisano E.D., Morris E.A., Weatherall P.T. (2016). Neoadjuvant chemotherapy for breast cancer: Functional tumor volume by MR imaging predicts recurrencefree survival-results from the ACRIN 6657/CALGB 150007 I-SPY 1 TRIAL. Radiology.

[17] Jahani N., Cohen E., Hsieh M.K., Weinstein S.P., Pantalone L., Davatzikos C., Kontos D. Deformable image registration as a tool to improve survival prediction after neoadjuvant chemotherapy for breast cancer: Results from the ACRIN 6657/I-SPY-1 trial. Proceedings of the Medical Imaging 2018: Computer-Aided Diagnosis.

[18] Jahani N., Cohen E., Hsieh M.-K., Weinstein S.P., Pantalone L., Hylton N., Newitt D., Davatzikos C., Kontos D. (2019). Prediction of Treatment Response to Neoadjuvant Chemotherapy for Breast Cancer via Early Changes in Tumor Heterogeneity Captured by DCE-MRI Registration. Sci. Rep..

[19] Olshen A., Wolf D., Jones E.F., Newitt D.C., Veer L.V., Yau C., Esserman L., Wulfkuhle J.D., Gallagher R.I., Singer L. (2017). Features of MRI stromal enhancement with neoadjuvant chemotherapy: A subgroup analysis of the ACRIN 6657/I-SPY TRIAL. J. Med. Imaging.

[20] LeCun Y., Bengio Y., Hinton G. (2015). Deep learning. Nature.

[21] Panigrahi S., Nanda A., Swarnkar T. (2009). A Survey on Transfer Learning. IEEE Trans. Knowl. Data Eng..

[22] Wang Z., Li M., Wang H., Jiang H., Yao Y., Zhang H., Xin J. (2019). Breast Cancer Detection Using Extreme Learning Machine Based on Feature Fusion with CNN Deep Features. IEEE Access.

[23] De Yu S., Liu L.L., Wang Z.Y., Dai G.Z., Xie Y.Q. (2019). Transferring deep neural networks for the differentiation of mammographic breast lesions. Sci. China Technol. Sci..

[24] Kumar N., Verma R., Arora A., Kumar A., Gupta S., Sethi A., Gann P.H. Convolutional neural networks for prostate cancer recurrence prediction. Proceedings of the Medical Imaging 2017: Digital Pathology.

[25] He Y., Guo J., Ding X., Van Ooijen P.M.A., Zhang Y., Chen A., Oudkerk M., Xie X. (2019). Convolutional neural network to predict the local recurrence of giant cell tumor of bone after curettage based on pre-surgery magnetic resonance images. Eur. Radiol..

[26] Liu M.Z., Mutasa S., Chang P., Siddique M., Jambawalikar S., Ha R. (2020). A novel CNN algorithm for pathological complete response prediction using an I-SPY TRIAL breast MRI database. Magn. Reson. Imaging.

[27] Ravichandran K., Braman N., Janowczyk A., Madabhushi A. A deep learning classifier for prediction of pathological complete response to neoadjuvant chemotherapy from baseline breast DCE-MRI. Proceedings of the Medical Imaging 2018: Computer-Aided Diagnosis.

[28] Wu J., Cao G., Sun X., Lee J., Rubin D.L., Napel S., Kurian A.W., Daniel B.L., Li R. (2018). Intratumoral spatial heterogeneity at perfusion MR imaging predicts recurrence-free survival in locally advanced breast cancer treated with neoadjuvant chemotherapy. Radiology.

[29] Esserman L.J., Berry D.A., Cheang M.C.U., Yau C., Perou C.M., Carey L., DeMichele A., Gray J.W., Conway-Dorsey K., Lenburg M.E. (2012). Chemotherapy response and recurrence-free survival in Neoadjuvant breast cancer depends on biomarker profiles: Results from the I-SPY 1 TRIAL (CALGB 150007/150012; ACRIN 6657). Breast Cancer Res. Treat..

[30] Newitt N., Hylton D. (2016). Multi-center breast DCE-MRI data and segmentations from patients in the I-SPY 1/ACRIN 6657 trials. Cancer Imaging Arch..

[31] Newitt N., Hylton D. (2016). Single site breast DCE-MRI data and segmentations from patients undergoing neoadjuvant chemotherapy. Cancer Imaging Arch..

[32] Partridge S.C., Gibbs J.E., Lu Y., Esserman L.J., Tripathy D., Wolverton D.S., Rugo H.S., Hwang E.S., Ewing C.A., Hylton N.M. (2005). MRI measurements of breast tumor volume predict response to neoadjuvant chemotherapy and recurrence-free survival. Am. J. Roentgenol..

[33] Clark K., Vendt B., Smith K., Freymann J., Kirby J., Koppel P., Moore S., Phillips S., Maffitt D., Pringle M. (2013). The cancer imaging archive (TCIA): Maintaining and operating a public information repository. J. Digit. Imaging.

[34] Esserman L.J., Berry D.A., DeMichele A., Carey L., Davis S.E., Buxton M., Hudis C., Gray J.W., Perou C.M., Yau C. (2012). Pathologic complete response predicts recurrence-free survival more effectively by cancer subset: Results from the I-SPY 1 TRIAL–CALGB 150007/150012, ACRIN 6657. J. Clin. Oncol..

[35] Russakovsky O., Deng J., Su H., Krause J., Satheesh S., Ma S., Huang Z., Karpathy A., Khosla A., Bernstein M. (2015). ImageNet Large Scale Visual Recognition Challenge. Int. J. Comput. Vis..

[36] Burges C.J. (1998). A Tutorial on Support Vector Machines for Pattern Recognition. Data Min. Knowl. Discov..

[37] Mencattini A., Di Giuseppe D., Comes M.C., Casti P., Corsi F., Bertani F.R., Ghibelli L., Businaro L., Di Natale C., Parrini M.C. (2020). Discovering the hidden messages within cell trajectories using a deep learning approach for in vitro evaluation of cancer drug treatments. Sci. Rep..

[38] Casti P., Mencattini A., Comes M.C., Callari G., Di Giuseppe D., Natoli S., Dauri M., Daprati E., Martinelli E. (2019). Calibration of Vision-Based Measurement of Pain Intensity with Multiple Expert Observers. IEEE Trans. Instrum. Meas..

[39] Salakhutdinov R., Tenenbaum J.B., Torralba A. (2013). Learning with Hierarchical-Deep Models. IEEE Trans. Pattern Anal. Mach. Intell..

[40] Zheng L., Zhao Y., Wang S., Wang J., Tian Q. (2016). Good Practice in CNN Feature Transfer. arXiv.

[41] Saeys Y., Inza I., Larrañaga P. (2007). A review of feature selection techniques in bioinformatics. Bioinformatics.

[42] Mann D.R., Whitney B.H. (1947). On a Test of Whether one of Two Random Variables is Stochastically Larger Larger than the other. Ann. Math. Stat..

[43] Breiman L. (2001). Random forests. Mach. Learn..

[44] Kalousis A., Prados J., Hilario M. (2007). Stability of feature selection algorithms: A study on high-dimensional spaces. Knowl. Inf. Syst..

[45] Křížek P., Kittler J., Hlaváč V. Improving stability of feature selection methods. Proceedings of the International Conference on Computer Analysis of Images and Patterns.

[46] Castelvecchi D. (2016). Can we open the black box of AI?. Nature News.

[47] Csáji B.C. (2001). Approximation with Artificial Neural Networks. MsC Thesis.

[48] Alain G., Bengio Y. (2016). Understanding intermediate layers using linear classifier probes. arXiv.

[49] Jacovi A., Hadash G., Kermany E., Carmeli B., Lavi O., Kour G., Berant J. (2019). Neural network gradient-based learning of black-box function interfaces. arXiv.

[50] La Forgia D., Fanizzi A., Campobasso F., Bellotti R., Didonna V., Lorusso V., Moschetta M., Massafra R., Tamborra P., Tangaro S. (2020). Radiomic analysis in contrast-enhanced spectral mammography for predicting breast cancer histological outcome. Diagnostics.

[51] Fanizzi A., Pomarico D., Paradiso A., Bove S., Diotiaiuti S., Didonna V., Giotta F., La Forgia D., Latorre A., Pastena M. (2021). Predicting of sentinel lymph node status in breast cancer patients with clinically negative nodes: A validation study. Cancers.

[52] Fanizzi A., Basile T.M., Losurdo L., Bellotti R., Bottigli U., Campobasso F., Didonna V., Fausto A., Massafra R., Tagliafico A. (2019). Ensemble discretewavelet transform and gray-level co-occurrence matrix for microcalcification cluster classification in digital mammography. Appl. Sci..

[53] Losurdo L., Basile T.M.A., Fanizzi A., Bellotti R., Bottigli U., Carbonara R., Dentamaro R., Diacono D., Didonna V., Lombardi A. (2018). A Gradient-Based Approach for Breast DCE-MRI Analysis. Biomed Res. Int..

[54] Bellotti R., Bagnasco S., Bottigli U., Castellano M., Cataldo R., Catanzariti E., Cerello P., Cheran S., De Carlo F., Delogu P. (2004). The MAGIC-5 project: Medical applications on a grid infrastructure connection. IEEE Nucl. Sci. Symp. Conf. Rec..

[55] Losurdo L., Fanizzi A., Basile T.M.A., Bellotti R., Bottigli U., Dentamaro R., Didonna V., Lorusso V., Massafra R., Tamborra P. (2019). Radiomics analysis on contrast-enhanced spectral mammography images for breast cancer diagnosis: A pilot study. Entropy.

[56] Fanizzi A., Basile T.M.A., Losurdo L., Bellotti R., Tangaro S., La Forgia D., Didonna V., Massafra R., Tamborra P., Moschetta M. (2017). Hough transform for microcalcification detection in digital mammograms. Appl. Digit. Image Process. XL.

[57] Cox D.R. (1972). Regression Models and Life-Tables. J. R. Stat. Soc. Ser. B.

[58] Breslow N.E. (1975). Analysis of Survival Data under the Proportional Hazards Model. Int. Stat. Rev..

[59] Cook N.R. (2008). Statistical evaluation of prognostic versus diagnostic models: Beyond the ROC curve. Clin. Chem..

